# Continuous and scalable polymer capsule processing for inertial fusion energy target shell fabrication using droplet microfluidics

**DOI:** 10.1038/s41598-017-06746-3

**Published:** 2017-07-24

**Authors:** Jin Li, Jack Lindley-Start, Adrian Porch, David Barrow

**Affiliations:** School of Engineering, Cardiff University, Cardiff, CF23 5PH United Kingdom

## Abstract

High specification, polymer capsules, to produce inertial fusion energy targets, were continuously fabricated using surfactant-free, inertial centralisation, and ultrafast polymerisation, in a scalable flow reactor. Laser-driven, inertial confinement fusion depends upon the interaction of high-energy lasers and hydrogen isotopes, contained within small, spherical and concentric target shells, causing a nuclear fusion reaction at ~150 M°C. Potentially, targets will be consumed at ~1 M per day per reactor, demanding a 5000x unit cost reduction to ~$0.20, and is a critical, key challenge. Experimentally, double emulsions were used as templates for capsule-shells, and were formed at 20 Hz, on a fluidic chip. Droplets were centralised in a dynamic flow, and their shapes both evaluated, and mathematically modeled, before subsequent shell solidification. The shells were photo-cured individually, on-the-fly, with precisely-actuated, millisecond-length (70 ms), uniform-intensity UV pulses, delivered through eight, radially orchestrated light-pipes. The near 100% yield rate of uniform shells had a minimum 99.0% concentricity and sphericity, and the solidification processing period was significantly reduced, over conventional batch methods. The data suggest the new possibility of a continuous, on-the-fly, IFE target fabrication process, employing sequential processing operations within a continuous enclosed duct system, which may include cryogenic fuel-filling, and shell curing, to produce ready-to-use IFE targets.

## Introduction

Inertial fusion energy (IFE) production facilities have been proposed^1, 2^ based on laser-driven inertial confinement fusion (ICF), and which derive some of their design criteria from the physics explored in stockpile stewardship research facilities, such as the National Ignition Facility in the USA^3^ and Laser Megajoule in France^4^. ICF depends upon the precise interaction of multiple, powerful lasers and frozen hydrogen isotopes contained within small, spherical and concentric target shells, ultimately causing a fusion reaction at ~150 M°C. Commercial fusion energy production will require ~1,000,000 fuel targets per day, per reactor vessel, at an estimated 5000x unit cost reduction to ~$0.20^5^. One design for viable IFE targets^6, 7^ (*ϕ* 0.5 mm–4 mm), essentially comprise spherical shells (50–100 um wall thickness) of a low density (~250 mg/cm^3^), with interconnected voids (each <1 um diameter), with extreme sphericity^8^ (>99.9%, <50 nm roughness variation), and a high degree of concentricity (>99.0%). Within this shell, a precise quantity of smooth (Ra < 1 um RMS), frozen, and homogenous, deuterium-tritium matrix is ‘layered’^9–12^. Target shells have been fabricated manually from a range of low-Z materials (e.g. beryllium, diamond, CH polymer)^13–24^, and used to explore the concept of inertial confinement fusion with lasers^25–28^. However, current production methods for such targets, are incompatible with the anticipated massive demand and unit cost requirement, due to the low yield rate of shells meeting the sphericity and concentricity specifications, the long fabrication processing times, and subsequent shell quality control characterisation. The very extreme morphological, generation and yield rate specifications, have not been met, and hence, the ability to scale up target fabrication, remains one of the key challenges for future IFE application.

Multiphase droplet microfluidics can enable the efficient generation of monodisperse, double emulsion (DE) droplets, which could represent ideal templates of the polymeric IFE target shells^28–31^. However, current droplet curing processes are invariably separated from the microfluidic devices, using off-chip, batch methods. Typically, the collected DE droplets are left in a flask (or a rotating barrel) containing carrier medium, and the shell phase may be cured by photopolymerisation. Ordinarily, the polymer is subject to 8–15% volumetric shrinkage, and experiences polymerisation stress during the solidification, which potentially gives rise to huge morphological distortions in the shells, when formed around an incompressible liquid mandrel. These issues led to the widely-adopted conclusion, that such polymer shells should be cured slowly by a mild process (low polymerisation stress generation, and long relaxation time), to attain a spherical and concentric form. In addition, it has been assumed that, the component liquid phases of DE droplets must be density matched^32^. This is generally achieved by using a non-reactive solvent^23^, so that the inner droplet could be more readily centralised within the DE droplet, by hydrodynamic (buoyancy) forces during polymerisation. These stringent conditions, render the shell fabrication process, with long fabrication durations (ranging from 60 minutes to several hours)^24^, relatively low yield rates of intact shells^17^, and polydisperse shell geometrical specifications^18^. These issues could be due, in part, to the heterogeneous polymerisation conditions for DE droplets, within the curing baths employed. It is also difficult to determine whether the cured shells result in a rigid final structure, as the high conversion degree of polymer occurs when initiated by free-radicals quickly^33^. Several reviews and publications have written about the on-flow fabrication of microparticles using microfluidics devices^34–38^. However, there has been no clear evaluation of the impact of the photocuring conditions, on a moving DE droplet, and the resulting shell geometry, with specific reference to the extreme sphericity and concentricity requirements for IFE application. Hence, it is difficult to identify the critical experimental parameters required for target shell production using channel microfluidics. It also limits both, the usage of additional functional components within the polymer, to enhance the mechanical properties of shells (e.g. functionalized carbon nanotubes, as shown in our previous work^39^).

**Figure 1:**
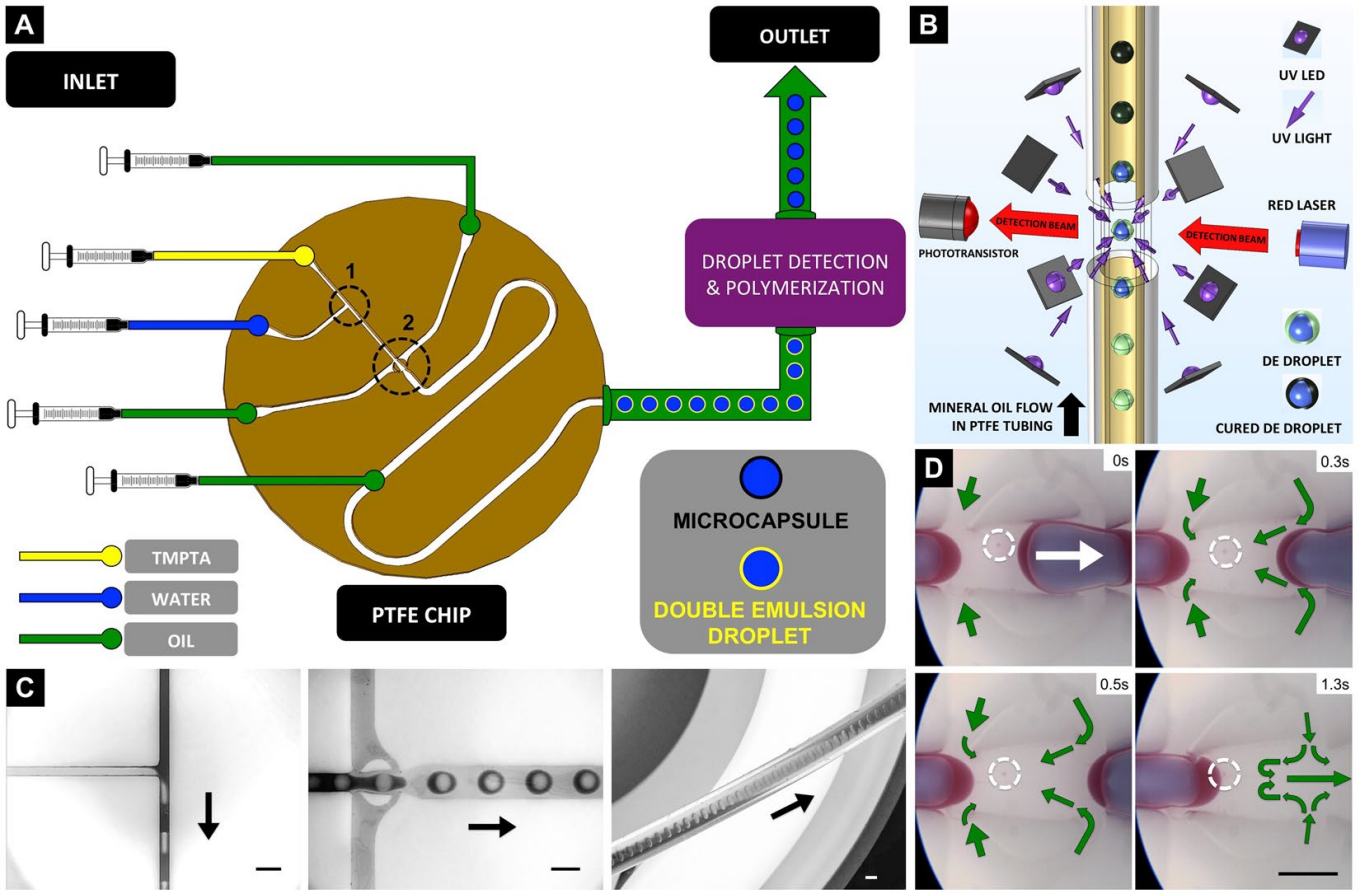
On-flow target shells fabrication system. (**A**) Schematic drawing of the experiment setup. Dotted circles 1 and 2 are a T-shaped droplet forming junction, and a ‘bat-wing’ droplet forming junction, respectively. **(B**) Schematic drawing of the on-flow DE droplet detection and UV curing process. **(C)** Water droplets were dispersed in the TMPTA phase at a T-shaped junction (left image). The core-shell shaped, liquid TMPTA segments, were sheared-off by the mineral oil flow at a bat-wing junction. (middle image) Monodispersed DE droplets flowed in a horizontal outlet tubing (right image). **(D**) Timesequenced images, show the removal of the satellite droplet at the bat-wing junction by merging with a subsequent dispersed phase. The green arrows indicate the direction of the mineral oil flow. All the scale bars are 1 mm.

In this paper, we test the notion of a new, continuous, enclosed microreactor-based, IFE target-shell processing operation, that has potential to scale up to meet future IFE generation demands. Our current operation (Fig. 1A,B) includes three procedures: (i) double emulsion droplet formation, (ii) on-flow double emulsion droplet centralisation, and (iii) on-flow ultrafast shell phase polymerisation. We evaluate and optimise each procedure, by experiment and numerical modeling, and provide new solutions for the preparation of precursors (no density matching processing required), the removal of satellite droplets, and the real-time analysis of double emulsion droplet shapes. We also investigate the relative advantages of different illumination regimes for photocuring, and compare these using optical microscopy, which although not a high resolution technique, is considered here, as a suitable comparative methodology. The attained shells attained over 99.0% sphericity and concentricity, at a near-100% yield rate.

## Results

### Monodispersed double emulsion droplet formation with satellite droplets removal

**Figure 2:**
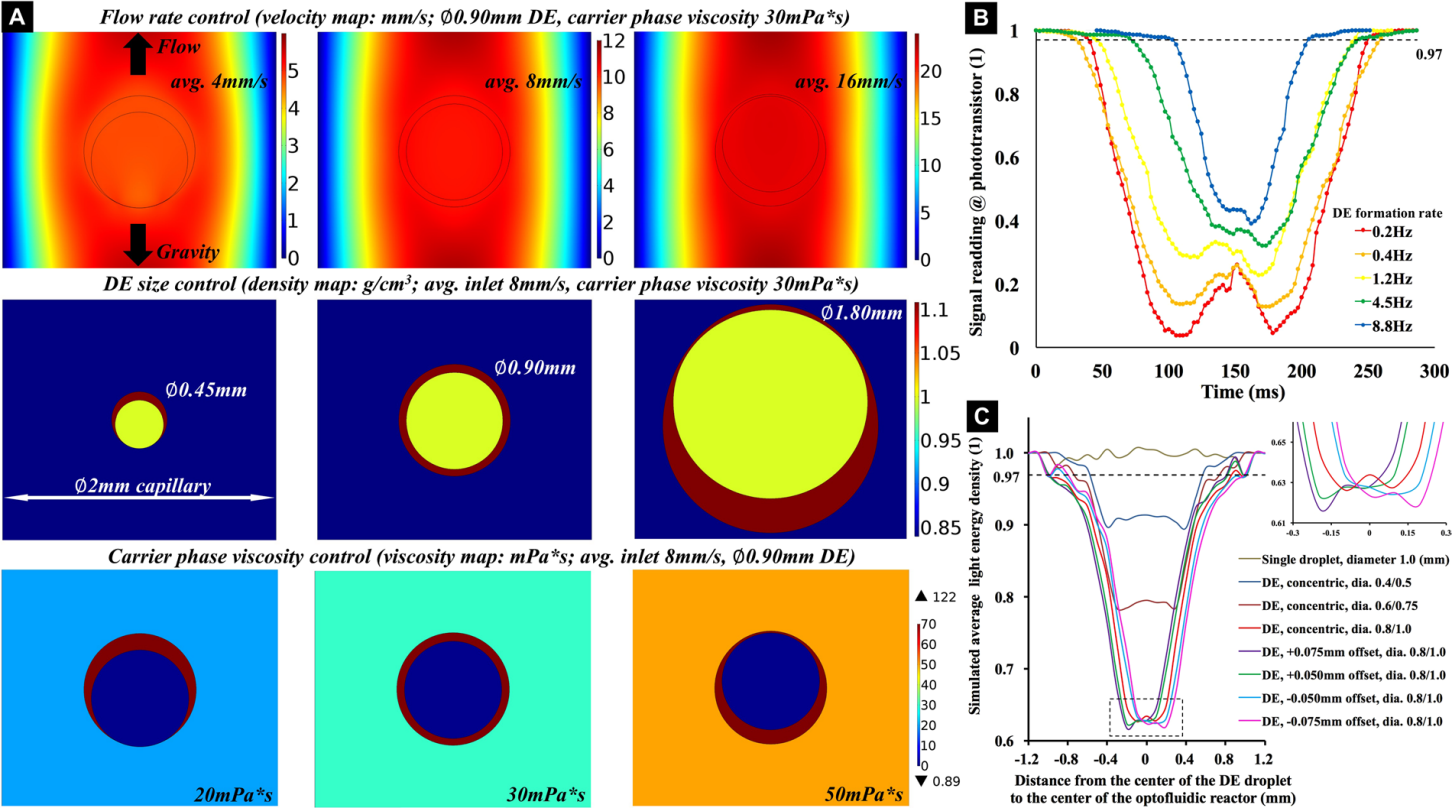
On-flow DE droplet centralization, detection and shape analysis. (**A**) Simulated images show that the equilibrium states of DE droplet shapes when the droplets are rising in 2 mm wide tubular capillary, with the presence of gravity. The states are influenced by the average flow velocity, the size of DE droplet and carrier phase velocity. While a φ 0.90 mm DE droplet flows in an avg. 8 mm/s laminar flow, carried by 30 mPa*s continuous phase, the droplet attains a spherical and concentric form. (**B**) The charts show the phototransistor detection signal readings of the rising DE droplets, which were formed at different rates. The presence of DE droplets diverged the red laser light, resulting in decreased incident irradiance on the phototransistor, compared to the nominal value obtained with a mineral oil (only) carrier phase flow. The signal shapes change from “W” to “V”, indicating different beam transmission situations. This was due to lensing effects from morphologies variation of DE droplets, when passing across the detection beam. **(C**) Simulated on-flow droplet detection signal, predicting that concentric DE droplets produce a “W” shaped photo-detector signal, non-concentric DE droplets produce a “V” shaped photo-detector signal, the magnitude of which depends upon the size of the DE droplet and the water droplet position. Highlighted section of upper image is shown in lower image in more detail.

Polymeric shells were produced from DE droplets, formed in a planar PTFE microfluidic chip, using pure liquids, without density balancing or surfactant additions. As shown in Fig. 1C, deionized water droplets were sheared off by the TMPTA flow, at a T-shaped fluidic junction. Then, core-shell shaped segments were dispersed in continuous mineral oil flow, at a ‘bat-wing’ junction (Fig. 1D). As this dispersed phase moved through the bat-wing fluidic junction^40^, the continuous phase, mineral oil flow, was confined by the junction structure. As the four, counter oil flows (indicated by the green arrows) met at the joint area, a ‘still’ zone was formed. Satellite droplets^41, 42^ retained a stationary position within the zone, and advantageously merged with subsequent droplets of dispersed phase (Fig. 2D and Movie S1). Two-phase, laminar flow, simulations of droplet formations were conducted, and revealed the details of the flow patterns (Fig. S2). This satellite droplet removal mechanism, improved the uniformity of the DE droplets, and is important for further on-flow droplet processing. This is because any satellite droplets, entering the outlet tubing, will accumulate over time, and interfere with the subsequent droplet detection and shell photocuring, by either (i) randomly merging and causing greater DE droplet polydispersity, (ii) adhering to the outlet tubing wall causing an interruption to the double emulsion flow, or (iii) acting as microscale spherical lenses which could interfere with the detection beam and the curing UV illumination. With the fluidic junction configurations, the radius of the DE droplets could be tuned from 350 to 1200 um diameter, by changing the inflow rates. Fig. S3 shows that the DE droplets could be produced consistently at up to 20 Hz, with 2.15% (n = 100) size variation (1.85% @ 7 Hz, n = 100). After the droplet forming junctions, another inlet was applied, to pump additional carrier phase mineral oil into the double emulsion. The DE stream was then passed to the optofluidic reactor, rising vertically through the LED housing. This verticality maintained droplet migration along the centerline of the flow, and avoided undesired contact between the DE droplets and the tubing wall.

### On-flow DE droplets centralization and shape detection

To fabricate solid polymeric shells with high geometry specifications for IFE targets, it is necessary to maximize the sphericity and concentricity of the DE droplets before the polymerisation of the shell phase, where the geometry would be fixed. In our experiments, since no surfactant was employed, the surface tension at the fluid interfaces, were not reduced, which maintained the moving DE droplets as spherical forms. The DE droplet centralisation was achieved in the vertically rising laminar flow, by tuning the inflow parameters. We found that several factors, including the flow velocity, the ratio of the DE droplet diameter to the capillary width, and the dynamic viscosity of the carrier phase, influenced the equilibrium state of the DE droplet shape, in the presence of the gravity. Informed by practical experiments, these flow conditions were optimised by subsequent mathematical modeling as shown in Fig. 2A. For a *ϕ* 0.9 mm DE droplet rising in a *ϕ* 2.0 mm tubular duct, its shape attained a spherical and concentric form, since the shear rate around the DE droplet is uniformly distributed, without any obvious trend of shape distortion (optimized for average linear flow velocity of 8 mm/s, and carrier phase dynamic viscosity of 30 mPa*s).

Separately, a droplet detection tool, which comprised a red laser emitter, a phototransistor and a microprocessor, was used to analyze the DE droplets shapes in real-time, before the photo-curing process. The emitter and the phototransistor were placed at the two ends of a ϕ 1.2 mm horizontal tubular channel, through the geometrical center of the optofluidic reactor (named optical focal point). Figure 2B shows the signal readings from the phototransistor, detecting the rising DE droplets, and which were generated at different frequencies from the droplet forming junction. From the low to high frequencies, the applied input flow rate combinations were 0.4/0.1/1.4/80, 0.8/0.2/1.8/80, 1.6/0.4/2.6/80, 3.2/0.8/3.2/100, and 6.4/1.6/4.5/120 (ml/hour) for the deionized water/TMPTA/continuous phase mineral oil/carrier phase mineral oil, respectively. As the total input flow rates were increased, it was found that the size of the signal decreased, and the shapes of valleys changed from a “W” shape to a “V” shape. These phenomena were studied by optical simulations, based on the practical experiments, as shown in Fig. 2C. The presence of spherical droplets at the optical focal point, diverge the incident detection beams, and form different energy patterns upon the receiving phototransistor. The details of the patterns, especially in z-axis, are influenced by the size of DE droplet and the variations of water droplet position within it, as shown in Fig. S4. When a concentric DE droplet rises vertically through the horizontal detection beam, a “W” shaped signal is shown, due to the equal lensing effects at both the polar regions of the DE droplet. In contrast, for a non-concentric DE droplet, the signal assumes a “V” shape, which itself depends upon the droplet morphology. In the subsequent practical experiments regarding this, the “-offset” conditions of the DE droplets at faster generation frequencies, can be explained by the reduction of DE droplet size, resulting from the higher shear stress of the faster flow at the first T-junction (whilst the water/TMPTA inflow ratio is kept constant). The shapes of DE droplets can be evaluated, to some extent, by the phototransistor detection signal, and those DE droplets of sufficient concentricity, as indicated by the most pronounced W-shaped phototransistor signal, were selectively photo-cured. The precision of this could be enhanced considerably, possibly by using light sheet microscopy, and new developments in interferometry.

### On-flow UV polymerization of capsule shells

**Figure 3:**
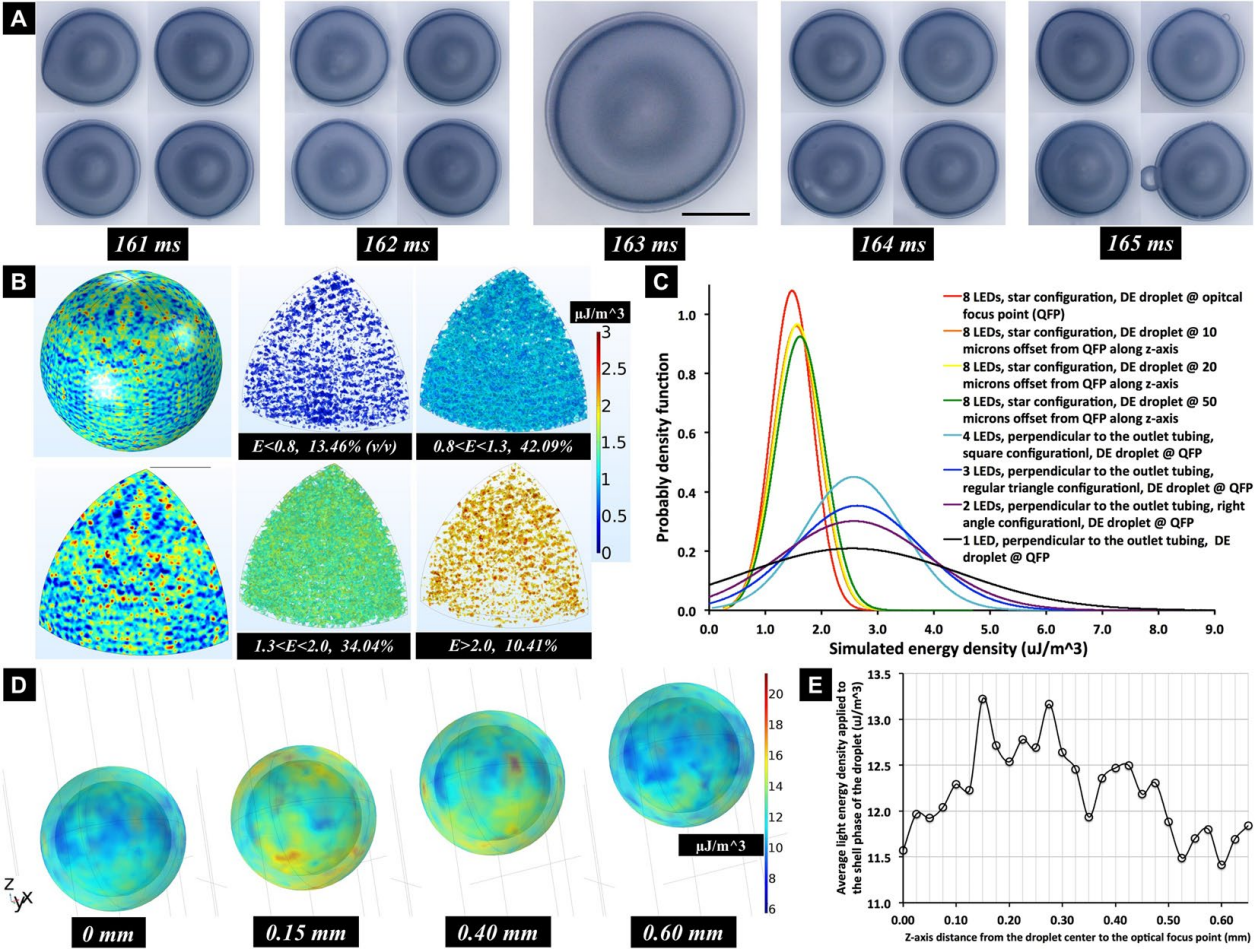
On-flow shell fabrication. (**A**) Images of the TMPTA capsule-shells, that were UV cured, under different delay times, *d*, between the initial droplet detection and the UV actuation. Scale bar denote 500 microns. (**B**) Simulation results of the UV light energy distribution within the shell phase of the *ϕ* 1 mm concentric and spherical double emulsion droplet, while it is placed at the reactor optical focal point. The right four images show the spatial distribution of different light energy densities (*E*) inside the shell phase, with their volume ratios relative to the whole droplet. **(C**) Probably density functions (n = 12,649 for each curve) of the simulated UV light energy densities, within the entire TMPTA shell, for different DE droplet locations, along the z-axis, on the centerline of the outlet tubing, as well as different LEDs configurations, indicate the uniformity of the UV irradiation upon the DE droplet. **(D**) Simulated UV light energy distribution upon the DE droplet, when it is rising in z-axis (flow direction) for 0.6 mm, during the 70 ms LEDs actuation period. **(E**) Graph of simulated average light energy density in the shell layer, when it is rising in z-axis, during the LEDs actuation period.

Current batch methodologies are used to slowly, but simultaneously, bake many DE IFE shells in a flask. Instead we designed an optofluidic reactor, to precisely photocure them individually, with programmable LED-actuation, whilst in a continuously flowing stream. This optofluidic reactor, comprised eight 365 nm UV light-emitting diodes (LED) as droplet-curing light sources, symmetrically mounted around the reactor, and were aligned with internal light pipes for the delivery of illumination. Preliminary experiments showed that illumination from a casually placed UV light source, led to highly non-uniform shells (Fig. S5), which were initially assumed to result from an uneven polymerisation rate over the shell phase. To reduce this assumed effect, the arrangement of light pipes within the optofluidic reactor was carefully devised by numerical simulation, to achieve a more uniform UV illumination, at its optical focus point. The actuation of the LEDs was controlled by the microprocessor, which was coupled to the droplet detector. The time of initial droplet detection was defined as, that moment when a consistent phototransistor reading drop, to 97% of the (default) pure mineral oil flow value (Fig. 2B, dotted line), and was registered. After a further delay, *d* (ms), the LEDs were temporarily actuated for an exposure duration, *T* (ms). The delay was applied to allow the droplet to be located at the center of the UV irradiation field, before the LEDs actuation (movie S2). We found that, by changing the value of the delay time *d*, with changes as low as 1 ms, the attained capsule-shells, surprisingly acquired significantly different globular forms. For instance, in one control experiment, concentric DE droplets were produced by the input flow rate combination of 0.5/0.1/1.4/80 (ml/hour) and flowed to the optofluidic reactor in the outlet tubing (inner *ϕ* = 2.16 mm). The UV LEDs were operated at their maximum current rating (0.85 A) and the exposure duration *T*, was set at 70 ms (~15 Hz). The delay time *d*, was the only variable changed, and its value was swept from 75 ms to 250 ms through 1 ms steps *(Δt* = 1 ms). When *d* equaled to 163 ms, the attained solidified capsule-shells (avg. outer diameter = 1529 ± 17 um(s.d.), avg inner diameter = 1386 ± 17 um(s.d.), avg. shell thickness = 71.64 ± 3.87 um(s.d.), avg. concentricity = 99.47%± 0.25 % (s.d.), avg. sphericity = 99.41%± 0.18% (s.d.), n = 50; optical system error = 2.5 um; measurement error = 0.16 um; measurement error was obtained by the repeat location and measurement of a single shell for 20 times), had an approximate spherical and concentric form, as shown in the central image of Fig. 3A. When *d* was in the range of 160 ms–162 ms or 164 ms–167 ms, unexpectedly, the shells acquired a very pear-shaped format, much like the rest of images in Fig. 3A. For the other values of *d*, the DE droplets did not result in continuous shells at all, but rather with circular openings within the resulting shell (inner phase leaking out) or, even cracking on the shells, as shown in Fig. S6. The variations in capsule-shell form were considered as the consequence of unequal UV light energy absorption, and resulting uneven photopolymerization, within the TMPTA shell.

### Simulations of the UV energy distribution upon the DE droplet

To investigate the photocuring process, ray trajectories of the UV light into the optofluidic reactor were simulated. With the DE droplet placed at the optical focus point of an eight LED reactor, the light energy distribution resulting from the accumulated UV irradiation, was relatively uniform within the shell phase (Fig. 3B-left). The other images show the spatial distribution of defined light energy densities (*E*), inside the shell phase, showing their volume ratios relative to the whole droplet. The uniformity of light energy within the TMPTA shell phase, was evaluated by the coordinate-based data of *E*, and is shown with the derived probably density functions. As shown in Fig. 3C,D, by moving the droplets away from the optical focal point, along the fluid flow direction (z-axis), the light energy distribution became more discretized, and more high energy spots would appear in the shell layer. As a comparison to the use of eight LEDs, other UV illumination conditions arising from different LED configurations (e.g. 4 LEDs), exhibited even more discretised light energy distributions in the shell phase, as shown in Fig. 3C and the movie S3. In addition, during the LEDs actuation period (70 ms), the total amount of UV light energy falling upon the shell layer, was a continuously changing quantity (Fig. 3E and movie S4), depending on the precise location of the DE droplet within the optofluidic reactor. We know from previous research, that these differences in light energy could result in the variation of photopolymerisation rate, within the TMPTA layer^43^, thereby causing spatially heterogeneous, volumetric shrinkage, and polymerisation stresses inside the curing TMPTA shells^44^. This would, therefore, predict the appearance of less spherical and concentric, cured plastic shells, initiated at specific reactor locations, where less homogenous illumination conditions prevailed, as shown in Fig. 3A (also in Figs S5 and 6). This is particularly the case, given that both the water mandrels and the oil carrier phase are essentially incompressible. Hence, in order to fabricate highly spherical and concentric polymer shells through photopolymerisation, the incident UV light needs to evenly deliver the photon energy to the entire shell phase matrix, to enable a uniform curing process. If the DE droplets are photocured continually, and on-the-fly, very short UV exposure times may be necessary to minimize the uneven energy absorption by the moving shell phase from the geometrically fixed, curing light sources.

### Inertial centralization of curing DE droplets in dynamic flow

**Figure 4:**
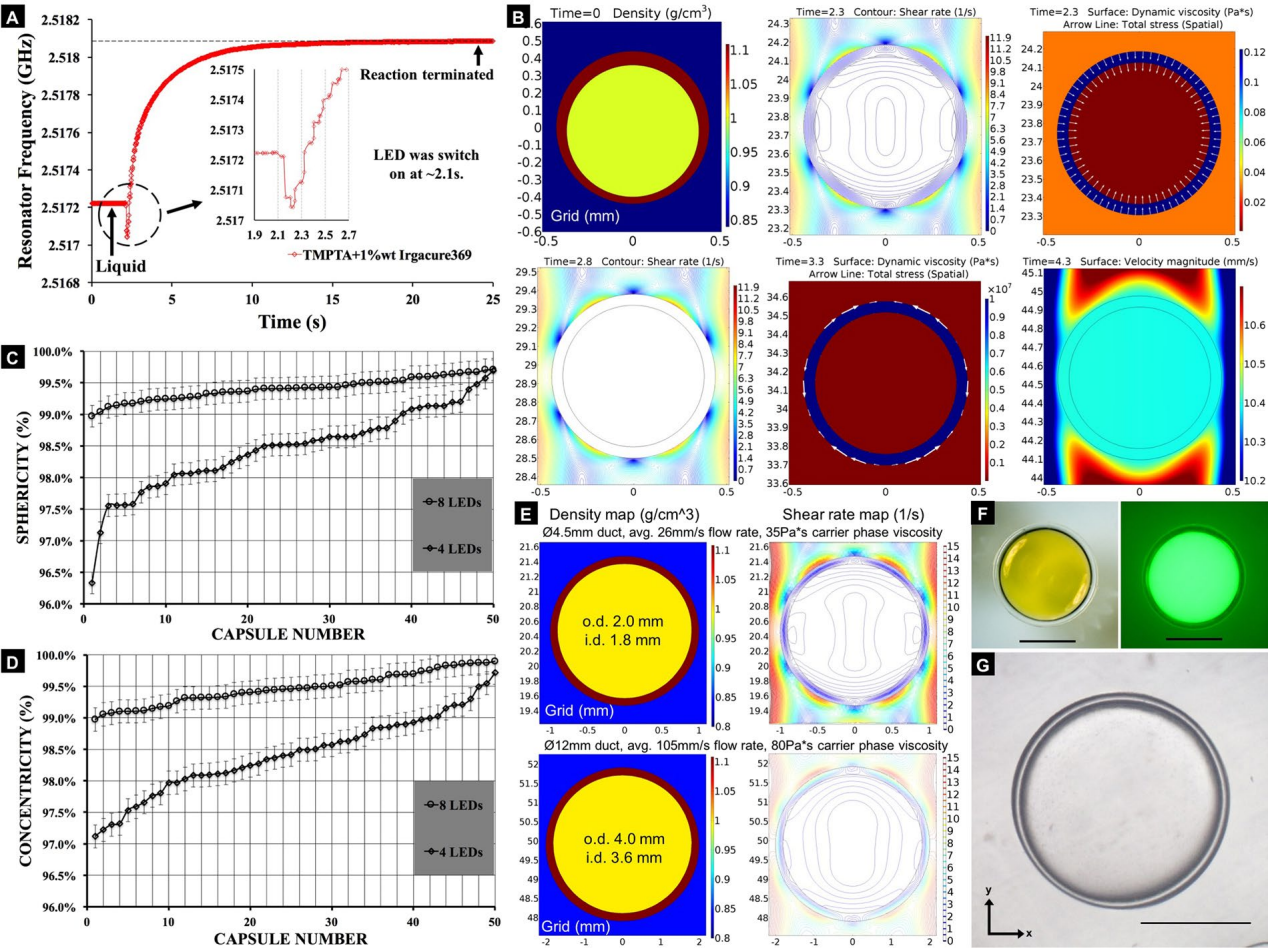
Inertial centralization of curing shells and shell evaluations. (**A**) Microwave resonator frequency changes over time, indicating changes in polymer permittivity. Experimental results obtained during photopolymerisation of TMPTA+ 1%wt Irgacure 369, held within a quartz capillary, inside the overall resonator. The UV LED was switched on for 5 second. (**B**) Simulated time sequence images of inertial centralization of DE droplet. Arrow lines are proportional to magnitude. T = 0s, density map, - offset DE droplet; T = 2.3s, shear rate map and viscosity map, centralized DE droplet, the viscosity of TMPTA is 0.122 mPa*s with inwards stress and symmetrical shear rate inside DE; T = 2.8s, shear rate map, shear rate inside DE is zero, while the viscosity is increased to ~0.5*106 Pa*s, T = 3.3s, viscosity map, the increase of TMPTA viscosity stopped at 1.0*107 Pa*s and the viscous stress only occurred at the oil/TMPTA interface in the normal direction of sphere tangent. T = 4.3s, centralized DE droplet, velocity magnitude map. (**C**,**D**) Charts of the rank-ordered sphericity and concentricity values, of the TMPTA capsule shells, cured by eight UV LEDs, in comparison to the others cured by four LEDs; average sphericity error = 0.17% and average concentricity error = 0.18%. (**E**) Simulated images of *ϕ* 2 mm (top row) and *ϕ* 4 mm (bottom row) double emulsion droplets attain concentric and spherical shape in different flow conditions, for the fabrication of different size IFE target shells. (**F**) Images of the TMPTA capsule shell around the core of deionized water with fluorescein sodium salt. Left, dark field image; and right, fluorescent image. (**F**) Confocal image (number of images = 40, z-axis step = 25 um) of TMTPA capsule shell, encapsulating a mixture of deionized water and glycerol, density matched to the TMPTA phase. All scale bars denote 500 microns.

The polymerisation kinetics of the TMPTA with 1 wt% Irgacure 369, both during the actuation of LED, and after its switch-off, was evaluated using polymer filled, quartz capillaries inside a ~2.5 GHz microwave resonator. This was to determine, non-invasively, any permittivity changes within the polymer, as an indication of the degree of curing. As shown in Fig. 4A, the results showed that, with the LED switched on for 5 s, the resonant frequency dropped initially, possibly due to the photoinitiator decomposition. The active radicals continuously crosslinked in the post-UV exposure environment, and the change in permittivity was almost complete after ~25 s. This indicates that to generate high quality polymeric IFE target shells within a continuously flowing stream, the optimal flow conditions required to maintain shells in a concentric and spherical form, must be maintained during the entire polymerisation process, and not just during photoinitiation.

At the onset of polymer viscosity change, Fig. 4B shows simulated time sequence images of a “-offset” DE droplet (at t = 0 s) that has been centralised (at t = 2.3 s), and then maintains that shape, while the dynamic viscosity of TMPTA changes from 122 mPa*s to 10^7^ Pa*s as a ramp function (between t = 2.3 s and t = 3.3 s. The dynamic viscosity of TMPTA during curing is about 1*10^5^ Pa*s, at the room temperature gelation point). This process indicated that the viscous force of the laminar flow, plus the gravitational force, centralized the moving DE droplet with symmetrical shear rates. Under these conditions, the total stress incurred at both the fluid interfaces, acts inwards, and is evenly distributed to retain the DE droplet sphericity. Given that the uniform UV irradiation is rapidly applied to the droplet, the photochemical reaction took place simultaneously throughout the entire TMPTA phase. While the TMPTA viscosity significantly increased, the shear rate was reduced to zero, and the velocity magnitude became uniform, rather than distributed as Poiseuille flow within the curing DE droplet (also shown in Fig. S7). The water mandrel experienced no offset, since the water and TMPTA phase had the same linear velocity. The stress at the oil-polymer interface was experienced in the direction of the sphere tangent, which was induced by the relative motion of the carrier phase to the curing shell. This also explained the visual observation in the practical experiments, that the cured capsules travelled slower than the DE droplets in the outlet tubing.

In terms of chemorheology, the ultrafast polymerisation used in our shell generation (70 ms UV pulse), would allow rapid propagation of active radicals, with more excess free volume, throughout the TMTPA phase^45^. This would lead to a higher degree of conversion, and hence a more rigid final shell structure, when compared to a slow initialisation. With respect to the conversion, the delay in shrinkage from using the faster photoinitiation, provides a longer, *elastic stress-free* state change^46^ in the developing molecular network, such that the original inwards stress at the fluid interfaces of the DE droplet (which tends to minimize the total surface area) constrains the direction of volumetric change. In contrast, the expected higher polymerization stress from rapid photoinitiation, is unlikely to cause shape deformation, since the TMPTA layer, is of a highly uniform thickness, and the stresses are evenly distributed throughout the entire spherical shell. This further indicates the importance of polymerisation uniformity, for high-specification polymeric shell fabrication, and that any spatial heterogeneity in the photoinitiation conditions, may cause permanent defects (Figs S5 and 6), even if the precursor DE droplets have attained the required concentricity and sphericity, before curing. In addition, it is worth noting that the use of a surfactant-free, double emulsions templates, may also have contributed to the enhanced concentricity and sphericity attained, since not only would surfactants reduce the inwards stress at the fluid interfaces, but they may also create concentration/heat gradients that could induce stress variations within the developing polymer molecular network. This could lead to possible shape deformations during the fast polymerisation. A similar argument could be laid against the use of additive reagents for density balancing.

The IFE target shell generation method demonstrated, is automated and in relative terms, highly replicable. As each of the monodispersed droplets were cured individually, under the same conditions, regular, and relatively spherical. Relatively concentric shells were formed, and the yield rate of contiguous shells, was near 100% (n = 1,000). Figure S8 shows images of 25 TMPTA capsule shells, that were fabricated and collected continuously, using the methods as described above. A group of control experiments was undertaken, whereby the eight LEDs, were replaced by four LEDs, which were positioned perpendicular to the outlet tubing, in order to create an optical focal point within the overall optofluidic reactor, but in a square format configuration. The concentricity and sphericity of shells attained using four LEDs, (concentricity = 98.43%± 0.62%; sphericity = 98.46%± 0.66%, n = 50) were found to have significantly lower values of both, sphericity (P = 6.37*10^−19^ < 0.05, one-tailed t-test) and concentricity (P = 2.10*10^−25^ < 0.05, one-tailed t-test), than those attained with eight LEDs (concentricity = 99.47%± 0.25%, error = 0.18%; sphericity = 99.41%± 0.18%, error = 0.17%; n = 50), as shown in Fig. 4C,D. These results correspond with the more heterogeneous, simulated illumination conditions, as shown in Fig. 3C. IFE targets shell specifications, vary between 0.5 mm and 4 mm in diameter. As shown in Fig. 4E, numerical simulations showed that both *ϕ* 2 mm and *ϕ* 4 mm double emulsion droplets, can be attained as spherical and concentric shapes, by changing the flow conditions. This indicates the possibility of producing shells for different dimensional requirements of the IFE targets, to serve the needs of different ICF reactors, using the on-flow fabrication methodologies presented here. Another group of control experiments were undertaken, by replacing the deionized water phase with (1) deionized water with fluorescent dye, and (2) a mixture of glycerol and deionized water, such that the inner phase density was matched with the shell phase. As shown in Fig. 4F,G, spherical and concentric TMPTA capsule shells, could be fabricated from the DE droplets, that were formed by these solvent mixtures, using the same DE droplet centralisation, on-flow detection and curing methods. This indicates that using these shell fabrication technique, density matching, may not be as important as others have found in their work.

## Discussion

The results demonstrate a new microfluidic-based operation, to continuously generate relatively spherical and concentric solid polymer capsule-shells for IFE target fabrication. This operation includes three main procedures. (I) Monodisperse double emulsion droplets were formed, without satellite droplets, in a planar microfluidic device, using surfactant-free templates, at up to 20 Hz. (II) The DE droplets were processed to attain concentric and spherical shapes, which were evaluated by a DE droplet detection tool, in a rising Poiseuille flow, before the shell solidification. (III) The concentric and spherical droplets, were photopolymerised individually, on-the-fly, by programmed, uniform-irradiation, 70 ms-length (~15 Hz) UV pulses, delivered from eight radially orchestrated light pipes, within an optofluidic reactor, without manual processing intervention. We found that the flow conditions, the timing and, critically, the uniformity of the light curing, all highly influenced the shell quality attained. The resultant ultrafast polymerization (70 ms), not only reduced the fabrication time, but also may improve the robustness of the shell, due to the high degree of polymer conversion obtained. The shapes of the attained capsules were relatively uniform, with near 100% yield rate, and both the sphericity and concentricity were over 99.0%, as measured by optical microscopy, albeit of limited accuracy and resolution for this specific demanding application. We also demonstrated by numerical modeling, that this overall method may be applicable to the production of shells of up to *ϕ* 4 mm, for a wide range of IFE applications.

One key issue that the results reveals, is the need for rapid, on-the-fly, high-resolution metrology of such ICF targets shells, most preferably within ducted, reactor systems. The coordinate-based, optical measurement equipment used here, had a system error of ~2.0035 um in the horizontal plane, and 0.5 um in the vertical-plane. The average measurement error was ~0.015% for both the sphericity and concentricity, which is insufficient for ascertaining the quality of high specification IFE targets shells. But, its use here, although insufficient to give precise, absolute dimensions, concerning low mode distortion over the shell surface, did allow the measurement of large numbers of shells, and the relative comparison of sphericity and concentricity, under different photo-curing regimes, which was the principle aim of the study. But for future work, much higher resolution measurement techniques are needed. Fast and extreme precision metrologies, such as interferometry, atomic force microscopy or tomography, have already been used for the characterization of cryogenic IFE target quality, and for other associated ICF experiments^47–50^. But, such metrologies are extremely time consuming and currently very difficult to scale up for use in mass-fabricated, IFE target, quality control. To solve this issue, further fabrication effort is required to enhance the precision uniformity, concentricity, and sphericity of target shells, not least in fluid delivery^51^, in a scalable fabrication process, in order to reduce the actual burden of shell quality characterisation. Equally, further developments are needed to enable precise, economical and non-invasive measurement methods^52–55^, to achieve rapid, on-line, quantitative data. Despite the limitations mentioned above, our work demonstrates that channel based, droplet microfluidics technology, can be applied to IFE target fabrication, not only to form monodisperse double emulsion droplets as templates for shell formation, but also for shell processing, including both the centralisation of DE droplets and their individual precision curing, which are difficult to achieve and automate, using conventional batch methods. Current effort is focused on (i) the generation of low-density foam, nanocomposite shells, using multiphase droplet microfluidics, (ii) precision fluidic delivery, and (iii) optofluidic reactors offering highly enhanced illumination uniformity and reduced pulse duration, to further improve shell quality. Such developments using enclosed, channel-based, fluidic systems, suggest the future possibility of enabling on-the-fly generation of IFE target shells, to include cryogenic permeation fuel-filling, and photo-cured, layered shell over-coating, to produce ready-to-use, high specification, IFE targets.

## Materials and Methods

### Material preparation and experiment setup

The TMPTA (PN 246808) and the inhibitor remover (PN 306312), oil red O (PN O0625), fluorescein sodium salt (PN F6377) and glycerol (PN G7893), were purchased from Sigma-Aldrich. After removal of monomethyl ether hydroquinone photo-inhibitor, the TMPTA was mixed with 1 wt% Irgacure 369 photoinitiator (BASF, peak absorption spectrum is at 366 nm wavelength), at 50 °C using a magnetic stirrer (RCT basic, IKA) for 1 hour, and subsequently kept, and used, in a dark environment. Deionized water, TMPTA, and mineral oil (PN M8410 Sigma-Aldrich) were degasified in a vacuum oven (Heraeus vacutherm, Kelvitron) before use. All the liquid phases were loaded into gas-tight, glass syringes (SGE Analytical Science) and injected into the microfluidic devices, through inlet TFE Teflon tubing (1.58 mm od, 0.8 mm id, SUPELCO) using displacement syringe pumps (789200 L, KD Scientific) to form DE droplets. The microfluidic channel configurations were machined on circular PTFE disks (50 mm diameter, 6 mm thickness) with a CNC milling machine (ProtoMat C30, LPKF), and the resultant surface roughness (Ra) measured using a VECCO NT3300 white-light interferometer, as 2 um. The disk was covered with a film of FEP (0.30 mm thickness, Goodfellows, UK) and sealed mechanically within stainless steel manifolds resulting in an enclosed fluidic channel system. The outlet Teflon tubing (3.2 mm od, 1.5 mm id, SUPELCO) passed vertically through the center of a LED housing, from the bottom, and its output placed in a glass container pre-filled with mineral oil. The LED housing of was made of powdered nylon (EOS, PA2200) and printed by a SLS 3D printer (EOS, P700). The 365 nm-wavelength UV LEDs (LZ1-00UV00, LED ENGIN) were symmetrically attached to the housing with rear-mounted heat sinks. All the LEDs were serially connected and powered with 0.85 A by Agilent E3633A DC power supplies. Activation of the UV LEDs was controlled by a microprocessor (Arduino Micro), coupled with a phototransistor (TEPT5700 110 InfraRed + Visible light, Vishay) that received 650–660 nm wavelength light from a red laser emitter (TLP-3200, TOSHIN CO., LTD).

The photocuring process of DE droplets were actuated after a consistent DE flow was observed inside the outlet tubing. The TMPTA capsule-shells were collected after the consistent flow was observed inside the outlet tubing. The TMPTA capsule-shells were measured in a petri dish filled with mineral oil. The TMPTA capsule-shells were washed and kept in acetone in a dark environment.

### Measurement

Real-time video was recorded to study the formation of the DE droplets using a high-speed camera (MS40KD2C1, Mega-speed) mounted on a Nikon AZ100 microscope. DE droplet formation frequencies at the bat-wing junction were obtained from the average value of the interval (n = 30) between the adjacent droplets breakup for each input flow rate combinations. Dimensional images of the DE droplets and the cured TMPTA capsule shells were captured using a Nikon MM-800 measurement microscope and analyzed with Nikon NIS-elements D 3.2 software. The sphericity and concentricity of the capsules were calculated with the following equations:

1 $$Sphericity=(1-\frac{{D}_{max}-{D}_{min}}{{D}_{avg}})\ast 100 \% ,\,and\,Concentricity=(1-\frac{{d}_{offset}}{{D}_{avg}}),$$

where D_max_, D_min_, and D_avg *avg*_ are the maximum, minimum, average capsule diameter, respectively, and derived from the measurement software; d_*offset*_ is the distance between the center of the outer circle and the center of the inner circle, of the capsule-shell.

### Simulations

COMSOL Multiphysics (version 5.2), laminar flow modules were used to simulate the TMPTA droplet breakup in mineral oil at the bat-wing junction, informed by the practical experiments. COMSOL Multiphysics (version 5.2), ray optics module was used to simulate the DE droplet detection, and the accumulated UV light energy distribution at the center of the optofluidic reactor by eight LEDs. Air, PTFE (tubing), mineral oil, liquid TMPTA, and water domains, and the reactor 3D model geometry, were defined as the practical experiment. UV rays was released at t = 0, to the eight light pipes (57,600 rays per light pipe, with total power 1.62E-3 W) at 45 degrees with the horizontal plane, to the optical focal point, using a 70-degree conical optical trajectory, as obtained from the LED optical characteristics datasheet. The developments of modeling were discussed and verified with COMSOL Multiphysics supporting team.

### Data and materials availability

All data needed to evaluate the conclusion in the manuscripts are present in the paper and/or the Supplementary Materials. Additional data related to this paper may be requested from the authors.

## Electronic supplementary material

Double emulsion droplets formation in microfluidic device

On flow droplet detection and polymerization

Simulated light energy distribution upon the droplet using different numbers of LEDs

Simulated light energy distribution upon the droplet while the droplet flows in the fluidic tubing

Continuous and scalable polymer capsule processing for inertial fusion energy target shell fabrication using droplet microfluidics
