# Supplementary material for: Continuous and scalable polymer capsule processing for inertial fusion energy target shell fabrication using droplet microfluidics
Source: Sci Rep. 2017 Jul 24;7:6302. doi: 10.1038/s41598-017-06746-3 (PMC5524715; doi:10.1038/s41598-017-06746-3)
Supplement: Supplementary file 5 — Continuous and scalable polymer capsule processing for inertial fusion energy target shell fabrication using droplet microfluidics [file 41598_2017_6746_MOESM5_ESM.docx]

**Continuous and scalable polymer capsule processing for inertial fusion energy target shell fabrication using droplet microfluidics**

Jin Li*, Jack Lindley-Start, Adrian Porch, & David Barrow

School of Engineering, Cardiff University, Cardiff CF23 5PH, United Kingdom

Correspondence and requests for materials should be addressed to Jin Li (email: LiJ40@cardiff.ac.uk)

**Supplementary figures**

**Fig. S1 (A)** The pratical experiment of microfluidic device. **(B)** Schematic drawing of mechanically-sealed, pressure-driven, multiphase microfluidic devices. **(C)** Image of the optofluidic reactor. The outlet tubing passed vertically through the center of the 3D printed LED housing (white). A 650-660nm red laser pen and a phototransistor (red wired), were aligned perpendicular to the outlet tubing, to detect droplet movement. Eight UV LEDs with heat sinks (four shown, in black, blue wired) were located symmetrically around the 3D printed unit to provide an optical focal point at its geometry center. **(D)** The inner geometry of the LED housing. The image shows the inner channels for the outlet tubing (vertical), the detection beam (horizontal), and the eight light pipes from which, UV illumination overlapped at the optical focal point of the reactor.


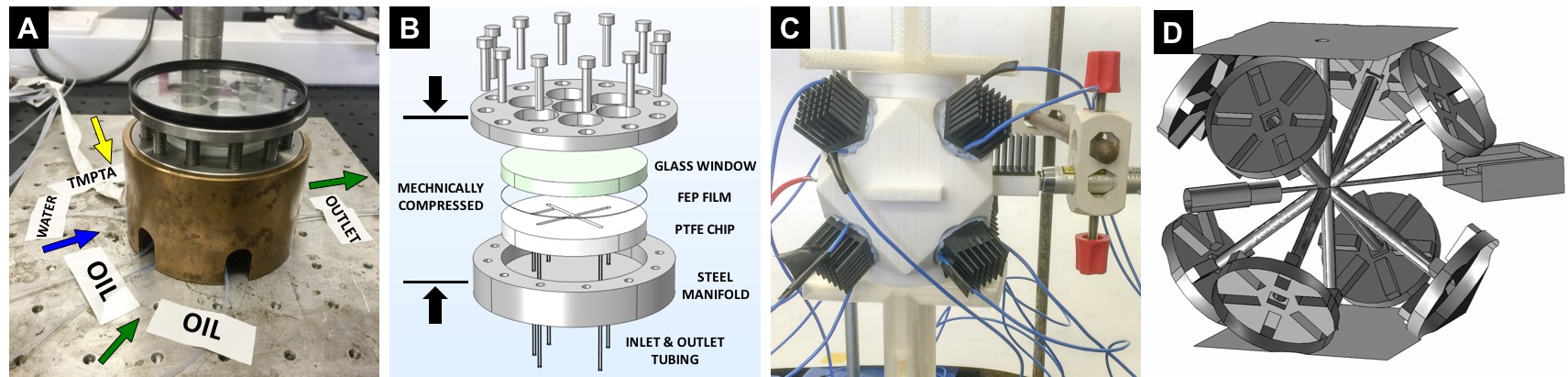

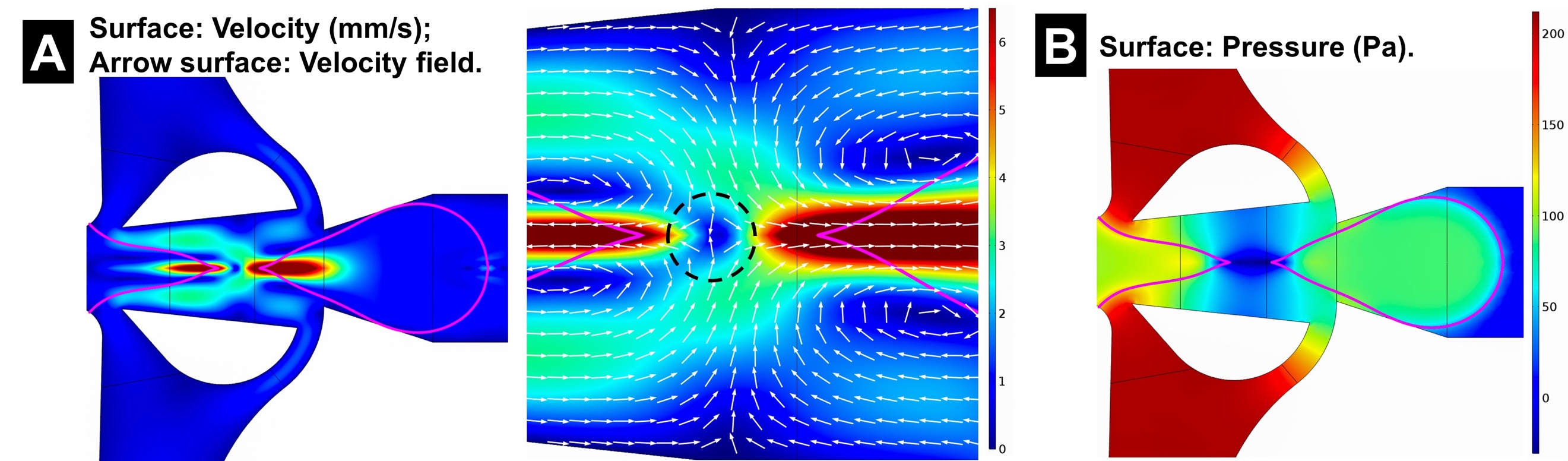


**Fig. S2** Simulated velocity field (A) and dynamic pressure distribution (B) of the TMPTA (boundary is highlighted by the magenta lines) droplet breakup in mineral oil at the bat-wing junction. The center of the black circle indicates a ‘still’ zone with very low velocity magnitude, which is to trap the satellite droplets.


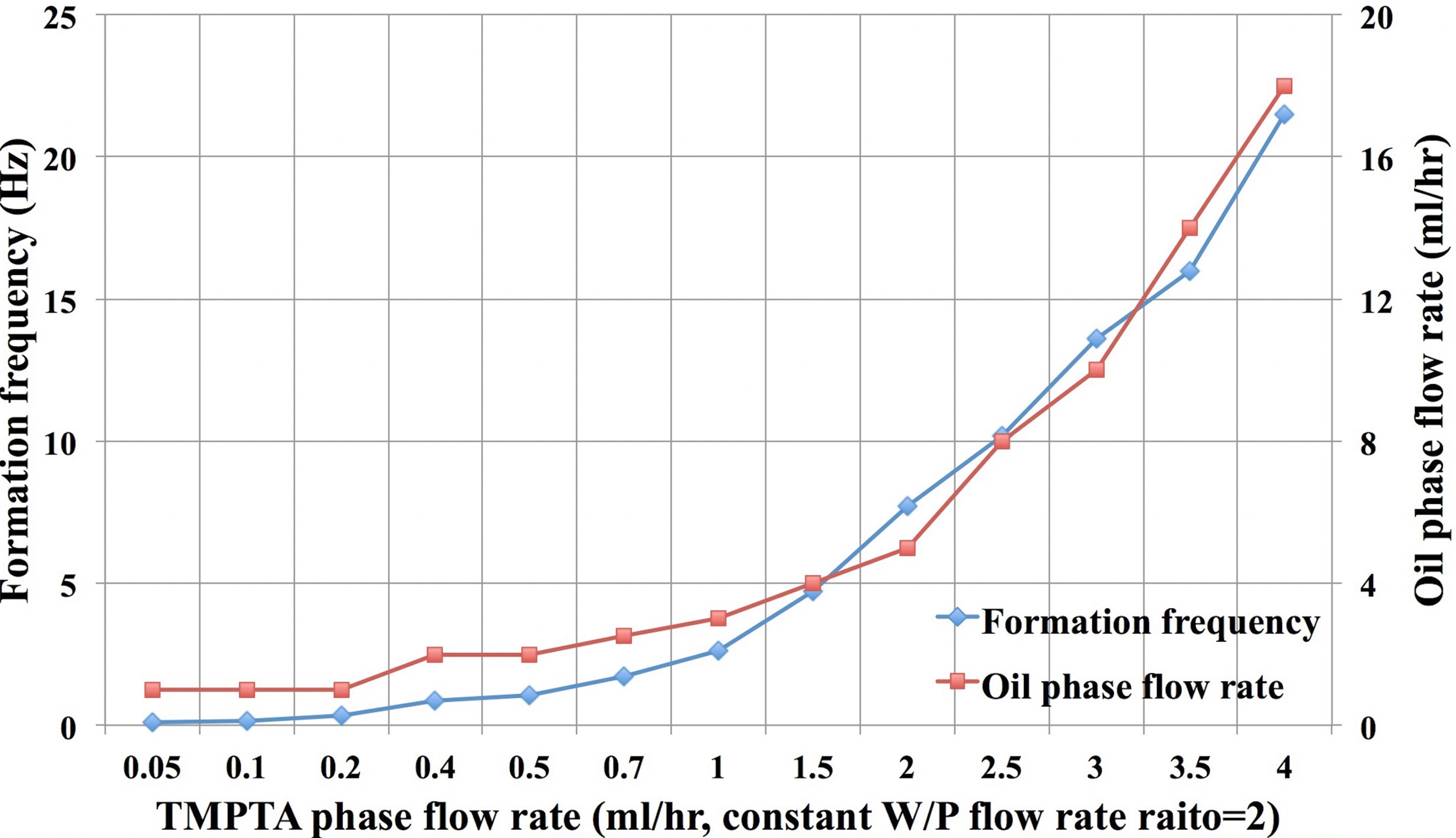


**Fig. S3** Graph of the DE droplet formation frequency (n=30, average error=0.82% for each data point) vs. TMPTA input flow rate vs. continuous phase mineral oil input flow rate.


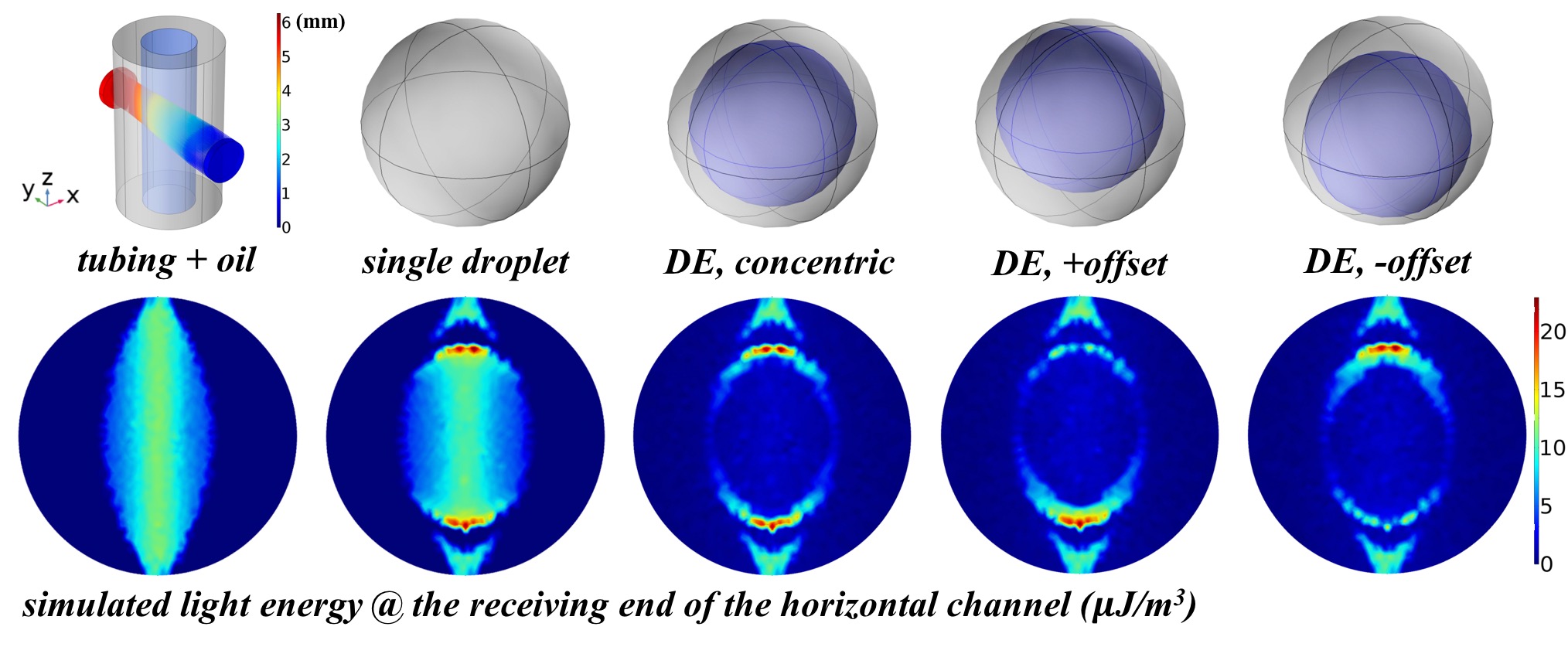


**Fig. S4** Simulated images of droplet detection. As the droplets are placed at the central of the beam, the energy patterns on the photo-detector change, due to ray divergence at the water/TMPTA interface.


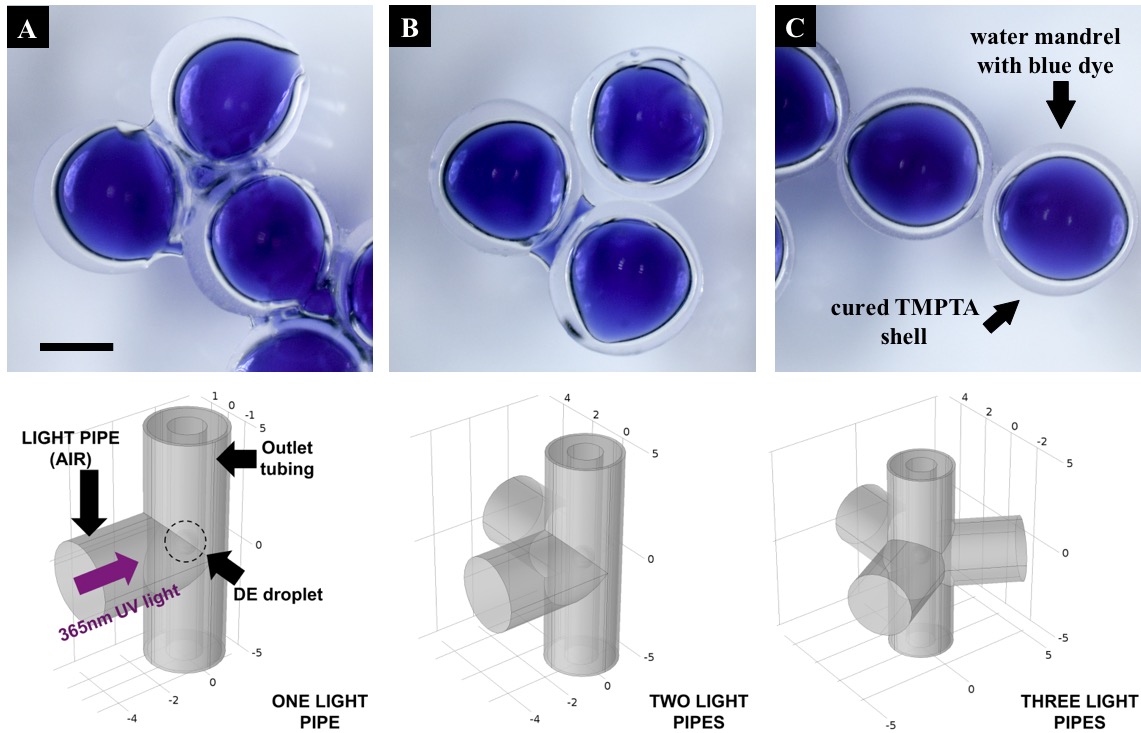


**Fig. S5. Images of non-intact TMPTA shells that were cured with one (A), two (B), and three (C) UV LEDs.** The scale bar is 500 microns. The schematic drawings below the images show the LEDs placements configurations for curing the shells.

**Fig. S6. Two main types of defect on the polymeric shell. (A)** Opening on the shell is resulted from curing the offset DE droplet. (**B)** Cracking on the surface are induced by the non-uniform polymerization stress over the shell.


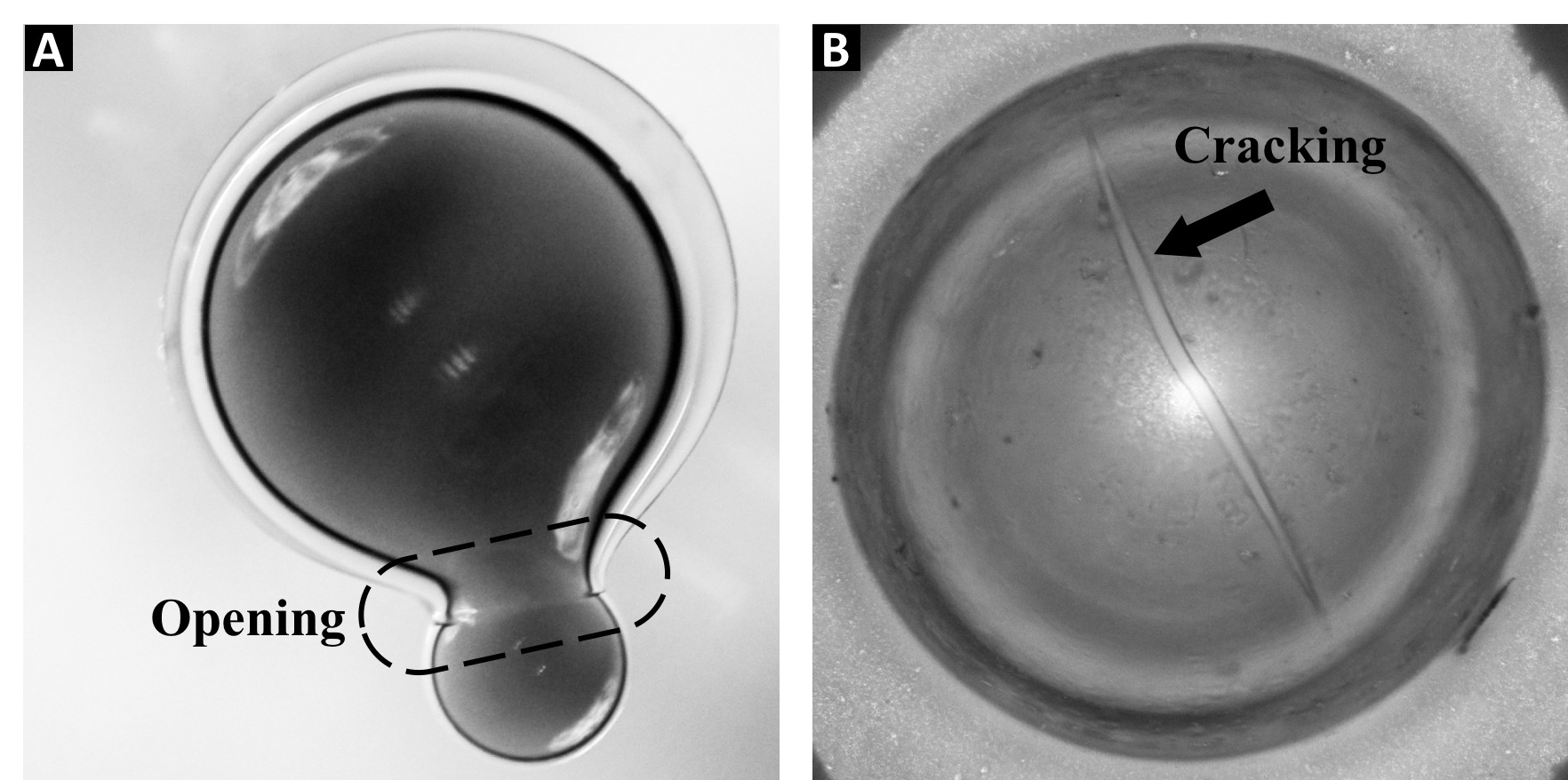

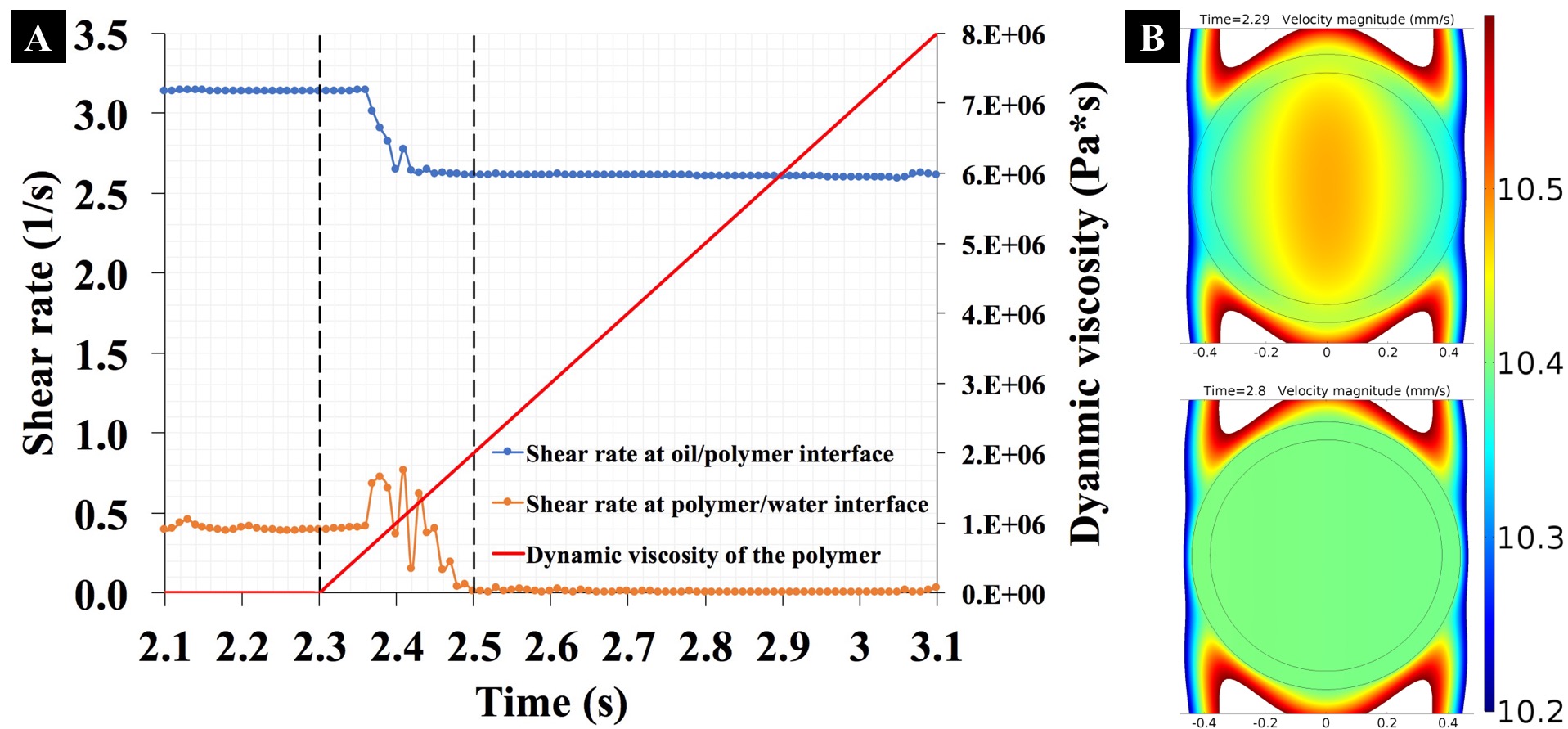


**Fig. S7. A.** Simulated result of the shear rate at the DE droplet interfaces in the rising laminar flow when the dynamic viscosity of TMPTA changes from 0.12Pa*s to 10^7^Pa*s. **B.** The velocity magnitude distribution inside the uncured DE droplet (top) and the curing DE droplets (bottom).

**Fig. S8. Images of intact TMPTA shells that were cured with our on-flow fabrication method.** The scale bar is 500 microns. **(A)** Cured shells rise in the outlet tubing. **(B)** TMPTA capsules in oil in a petri dish. **(C)** Images of 25 TMPTA capsule shells, that were collected contiguously, from the outlet. Formation materials of these capsule shells are deionized water, TMPTA with 1 wt% Irgacure 369, and mineral oil.


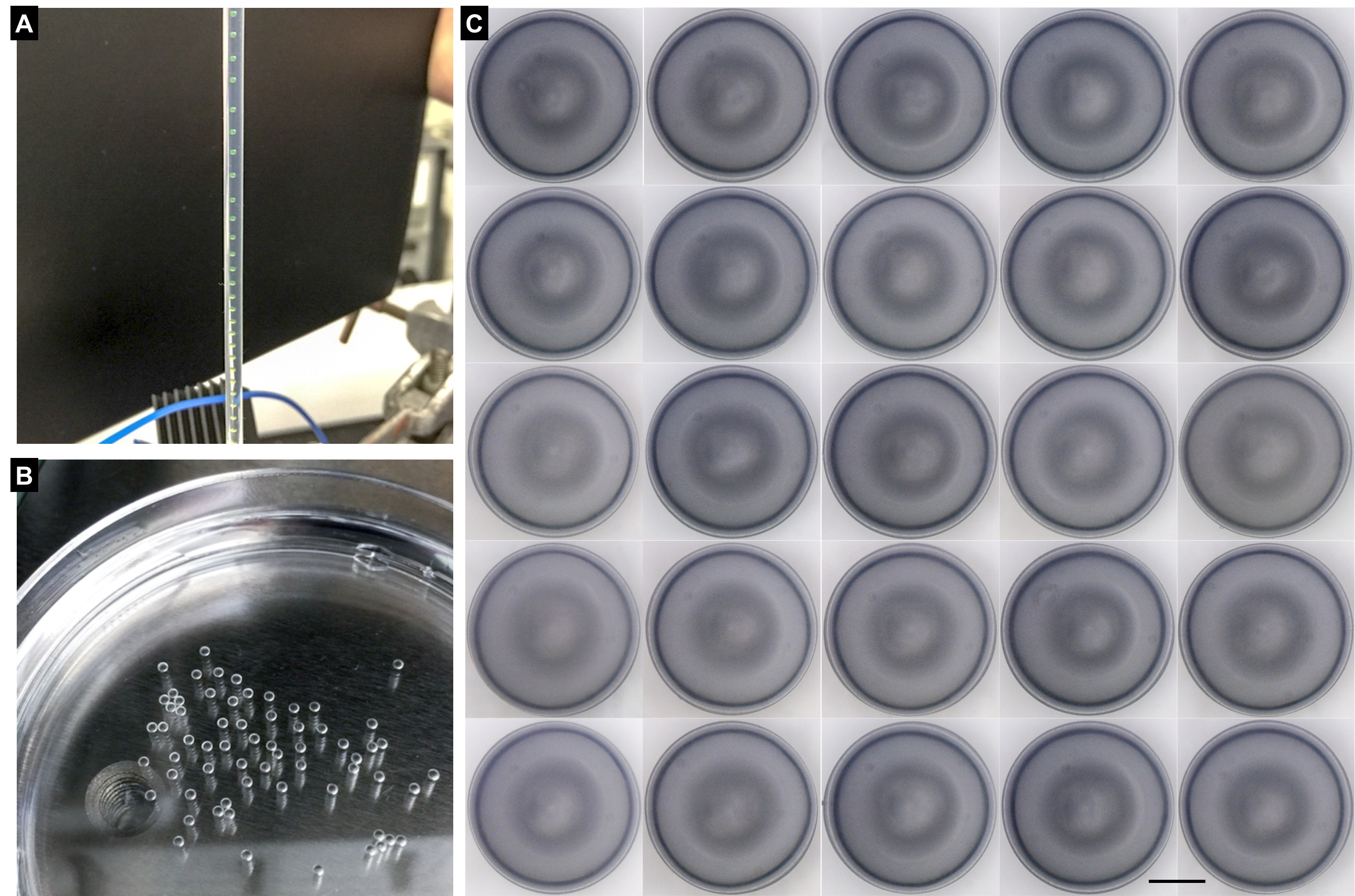


Supplementary video

**Supplementary video_1** Double emulsion droplets formation in microfluidic devices.

**Supplementary video_2** On flow droplet detection and polymerization.

**Supplementary video_3** Simulated light energy distribution upon the droplet using different numbers of LEDS.

**Supplementary video_4** Simulated light energy distribution in the droplet while the droplet flows in the fluidic tubing.
